# A coupled iron-hypoxia selection model for the evolution of endometriosis-associated ovarian clear cell carcinoma

**DOI:** 10.3389/fonc.2026.1965365

**Published:** 2026-09-16

**Authors:** Yasushi Umezaki, Ryoichi Okuma, Asako Fukuda, Yu Kamishita, Makiko Kurihara, Yukine Satomi, Kaoru Okugawa

**Affiliations:** Department of Obstetrics and Gynecology, Faculty of Medicine, Saga University, Saga, Japan

**Keywords:** ARID1A, clonal selection, endometriosis, ferroptosis, hypoxia, iron metabolism, labile iron pool, ovarian clear cell carcinoma

## Abstract

**Introduction:**

Ovarian clear cell carcinoma (OCCC) frequently arises in association with endometriosis and is characterized by iron-rich oxidative stress, hypoxia-responsive metabolic reprogramming, glycogen accumulation, and alterations in ferroptosis-regulatory pathways. Interactions among hypoxia, iron metabolism, oxidative stress, and ferroptosis are increasingly recognized, but their role during precursor evolution remains uncertain.

**Hypothesis:**

We propose that chronic iron-rich oxidative stress and a hypoxic microenvironment may jointly alter the relative fitness of epithelial cells during the endometriosis-to-OCCC transition. Repeated exposure may create a putative ferroptosis-related selection filter that preferentially removes susceptible cells while enriching less-susceptible cellular subpopulations capable of sustained survival. Here, ferroptosis tolerance denotes relative survival under chronic or sublethal ferroptosis-related stress and does not imply experimentally demonstrated resistance; broader oxidative-stress adaptation alone is insufficient to establish it. Surviving populations may acquire an adaptive iron-handling state that constrains the metabolically available labile iron pool despite an iron-rich extracellular environment through changes in iron uptake, ferritin-mediated sequestration and turnover, mitochondrial utilization, cellular export, or extracellular-vesicle-mediated ferritin disposal. These mechanisms may cooperate with HIF-1α/SLC1A1-associated adaptation and NRF2/SLC7A11/GPX4/glutathione pathways. Loss-of-function alteration of *ARID1A* may contribute to oncogenic evolution, while ARID1A deficiency may also modify redox and ferroptotic vulnerability and thereby the intensity of selection.

**Discussion:**

The novelty of this framework lies not in any single molecular interaction, but in applying interconnected iron, hypoxia, redox, and ferroptosis biology to an evolutionary selection model across the endometriosis–atypical endometriosis–OCCC continuum. The model predicts coordinated stress and iron-handling adaptations in precursor lesions, reproducible changes in population composition after repeated sublethal iron–hypoxia exposure, and, as a distinct outcome, durable phenotypic adaptation after washout. These predictions can be tested through cyst-fluid analysis, spatial profiling, and repeated-exposure, washout, rechallenge, and lineage-tracing experiments. The framework is a falsifiable hypothesis that may be supported, narrowed, reformulated, or rejected experimentally.

## 1 Introduction

Ovarian clear cell carcinoma (OCCC) is a distinct histological subtype of epithelial ovarian cancer and is particularly prevalent in Japan, where a national registry study reported that it accounted for nearly 30% of epithelial ovarian cancers (1). OCCC is characterized by relative resistance to platinum-based chemotherapy and by recurrent molecular alterations involving *ARID1A*, *PIK3CA*, the hepatocyte nuclear factor 1β (HNF-1β)-associated metabolic program, and the mechanistic target of rapamycin (mTOR) pathway (2, 3). Its frequent association with ovarian endometriosis provides an opportunity to examine carcinogenesis across a recognizable pathological continuum from typical endometriosis through atypical endometriosis to invasive carcinoma (3, 4).

A defining feature of ovarian endometriotic cysts is repeated hemorrhage into an enclosed, poorly drained cavity. Prolonged retention and breakdown of erythrocytes generate an iron-rich microenvironment capable of catalyzing reactive oxygen species formation, lipid peroxidation, and oxidative DNA damage (4–7). This has supported a predominantly injury-centered model in which iron-induced oxidative damage promotes mutagenesis and malignant transformation. That model is important but incomplete because it does not explain why a small subset of epithelial cells persist for years in a chemically hostile environment or how persistent survival might shape the phenotype of the eventual carcinoma.

Ferroptosis is a regulated form of cell death driven by iron-dependent phospholipid peroxidation and opposed by protective systems that include glutathione peroxidase 4 (GPX4), glutathione metabolism, lipid remodeling, and iron homeostasis (8–10). The iron-rich environment of endometriotic cysts therefore creates a biologically plausible setting in which ferroptosis-related stress could influence cellular survival. Hypoxia adds a second layer of complexity. Hypoxia-inducible factor 1α (HIF-1α) can promote metabolic adaptation and ferroptosis resistance through glutamate handling associated with solute carrier family 1 member 1 (SLC1A1) in some tumor systems (11), and HIF-1α and glucose transporter 1 (GLUT1) expression increase during OCCC development (12). However, hypoxia is not uniformly protective. Depending on cell type, duration, nutrient availability, and signaling context, it may promote adaptation, growth arrest, or cell death. Its contribution to survival in endometriotic precursor epithelium therefore remains a hypothesis rather than an established mechanism.

Evidence from endometriosis and ovarian cancer supports biological intersections among iron regulation, hypoxia, and ferroptosis. A small study of endometriotic cysts reported high cyst-fluid free iron, low oxygen tension, iron deposition, and carbonic anhydrase 9 (CA9) expression, and further suggested that hypoxia may perturb cellular iron regulation through stabilization of iron regulatory protein 2 (13). Endometriosis-associated mesenchymal stromal cells can support OCCC through iron regulation (14), HIF-2α has been implicated in established OCCC biology (15), and iron-overload-induced ferroptosis has been demonstrated in endometrial stromal cell models (16, 17). These studies establish plausibility but do not demonstrate that epithelial precursor cells undergo chronic iron–hypoxia selection before malignant transformation.

Recent spatial transcriptomic profiling with single-cell RNA-sequencing integration of tissues containing coexisting endometriosis and OCCC identified shared molecular features and pathways related to tumor progression, invasion, and metabolic reprogramming, supporting a transcriptomic continuum between the two compartments (18). Because the analysis was cross-sectional and included established carcinoma, it does not determine whether iron–hypoxia stress caused differential epithelial survival during precursor evolution.

The individual interactions among hypoxia, oxidative stress, iron metabolism, and ferroptosis are already recognized in multiple biological systems. The unresolved question is temporal and evolutionary: do these interconnected processes alter the relative fitness of epithelial populations during the precursor stage of endometriosis-associated OCCC? We propose a model in which persistent iron-rich oxidative stress and hypoxia jointly shape a selection landscape within endometriotic cysts. Ferroptosis is positioned as a candidate, but not necessarily exclusive, mechanism of selective cell loss. Surviving populations are predicted to acquire antioxidant, iron-handling, and metabolic adaptations that can persist or become enriched before invasive carcinoma develops. The specific contribution of this article is therefore the application of established and emerging biology to a falsifiable evolutionary model across the endometriosis–atypical endometriosis–OCCC continuum, rather than the claim that these molecular processes have previously been viewed as wholly independent.

## 2 Central hypothesis

We propose that OCCC can emerge through an evolutionary process in which epithelial cells within endometriotic cysts are repeatedly exposed to coexisting iron-rich oxidative stress and hypoxia. In this framework, genetic and epigenetic alterations generate cellular diversity, while the local environment influences which cells are lost, which survive, and which subsequently expand. The term coupled indicates that iron-related stress and hypoxia coexist within the same ecological niche and may jointly shape cellular fitness. It does not presuppose a supra-additive molecular interaction; synergy, additivity, and context-dependent antagonism remain experimentally distinguishable possibilities. Operational testing should therefore use a 2 × 2 factorial design that estimates the contributions of iron, oxygen context, and their interaction rather than assuming that combined exposure must be maximally cytotoxic.

### 2.1 Operational definitions and evidentiary boundary

Selection pressure is used here in an evolutionary sense to denote an environmental condition that produces reproducible differences in survival or expansion among heterogeneous cellular populations. Acute cytotoxicity alone is not evidence of selection. Operational evidence for selection would require repeated exposure accompanied by reproducible enrichment or depletion of identifiable clones or cellular populations attributable to differential survival or expansion, rather than state switching alone. Persistent phenotypic remodeling after washout may provide evidence of durable adaptation but, in the absence of differential enrichment, would not by itself establish selection.

Ferroptosis resistance denotes an experimentally demonstrated reduction in ferroptotic cell death under defined inducing conditions, supported by evidence of iron dependence, lipid peroxidation, and pharmacological or genetic rescue. Ferroptosis tolerance is used more cautiously to describe relative survival during chronic or sublethal ferroptosis-related stress without implying complete resistance, a fixed heritable trait, or dependence on a single protective mechanism. Oxidative-stress adaptation is broader and refers to the capacity to withstand reactive oxygen species and oxidative injury; it does not, by itself, establish ferroptosis tolerance.

Accordingly, the proposed ferroptosis-related selection filter is explicitly inferential. Direct ferroptotic elimination of susceptible epithelial cells, a joint contribution of iron-rich stress and hypoxia to selection, and stable enrichment of ferroptosis-tolerant populations have not been demonstrated in human endometriosis-associated precursor lesions. The model also does not assume that ferroptosis is the sole mechanism of cell loss. Apoptosis, necrosis, other regulated cell-death pathways, senescence, and proliferative arrest may contribute to the same ecological bottleneck.

### 2.2 Proposed evolutionary sequence

The model comprises five linked stages (**Figure 1**). First, repeated hemorrhage and prolonged retention of hemolyzed blood create sustained iron exposure and oxidative injury. Second, restricted oxygen availability and hypoxia-responsive signaling coexist with this iron-rich environment. Third, the combined stress landscape differentially affects epithelial cells, with ferroptosis proposed as one candidate mechanism of selective loss. Fourth, surviving populations constrain lethal lipid peroxidation through variable combinations of antioxidant defense, labile-iron regulation, and metabolic adaptation. Fifth, repeated differential survival or expansion changes population composition by enriching identifiable clones or cellular states; durable remodeling without differential enrichment would instead indicate persistent adaptation.

**Figure 1:**
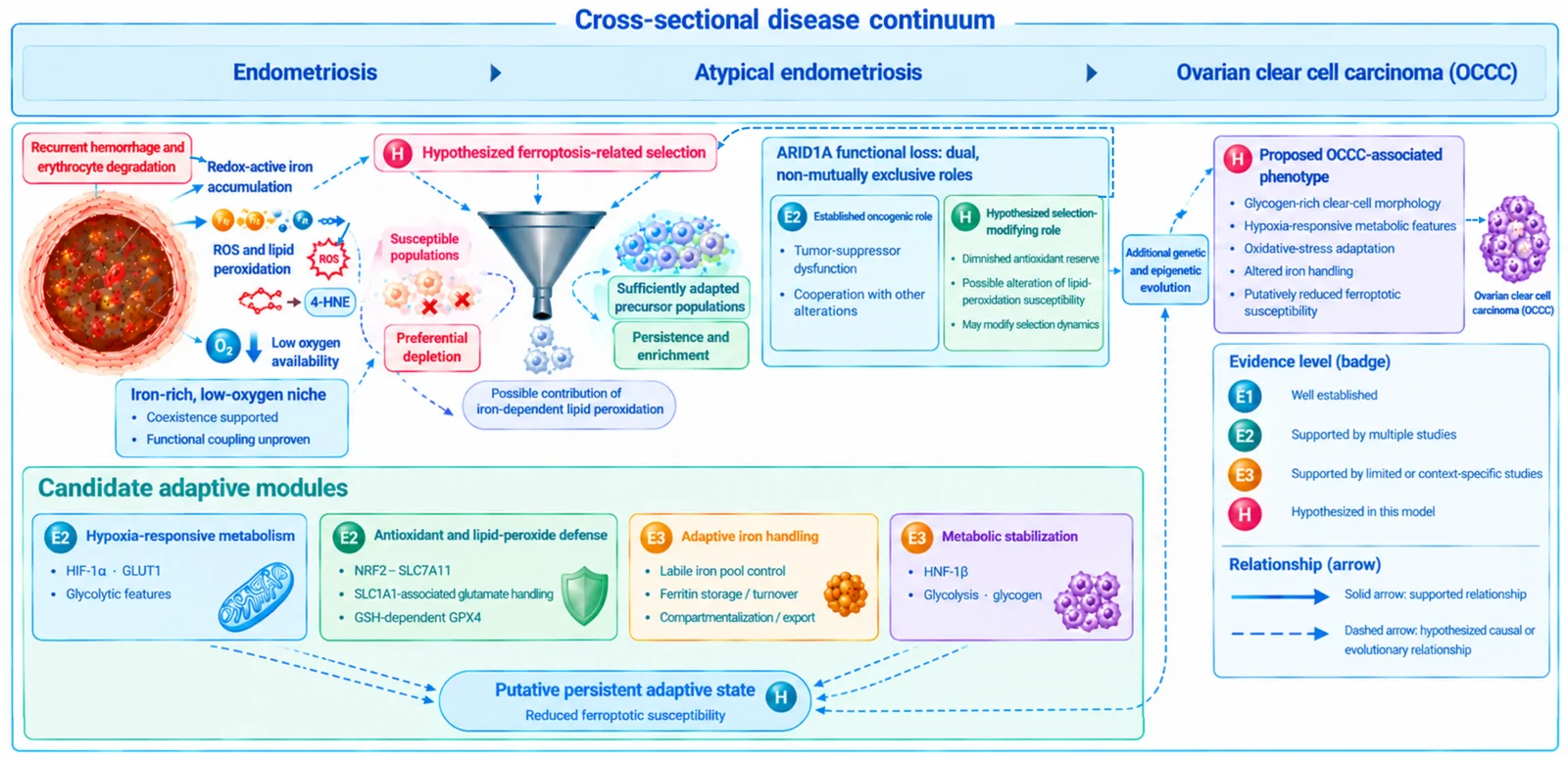
Evidence-stratified model of coupled iron–hypoxia selection during the evolution of ovarian clear cell carcinoma. The image summarizes the proposed progression from endometriosis through atypical endometriosis to ovarian clear cell carcinoma (OCCC). Repeated hemorrhage and retention of hemolyzed cyst contents create an iron-rich, lipid-peroxidative environment, while the enclosed cystic microenvironment is associated with hypoxia-responsive signaling. Their coexistence is supported, but their functional coupling as an epithelial selection pressure has not been directly demonstrated. The model proposes that persistent combined stress may create a putative ferroptosis-related selection filter. Ferroptosis is shown as a candidate, but not necessarily exclusive, mechanism of selective cell loss. Surviving populations may activate antioxidant pathways and acquire adaptive iron-handling states that constrain the metabolically available labile iron pool through sequestration, altered turnover, cellular export, extracellular-vesicle-mediated ferritin disposal, or other mechanisms. *ARID1A* alteration and loss of ARID1A function are depicted as established oncogenic driver events. ARID1A deficiency is additionally shown as a putative modifier of redox and ferroptotic vulnerability; this selection-modifying role remains unverified in human precursor lesions. Solid arrows indicate relationships supported in the relevant biological systems. Dashed arrows indicate predicted causal, temporal, or evolutionary relationships that require direct validation. The evidence coding does not imply that the complete sequence has been experimentally demonstrated in human endometriosis-associated OCCC development.

The hypothesis does not require every tumor to use an identical adaptive route. One population may depend predominantly on an antioxidant axis involving solute carrier family 7 member 11 (SLC7A11), GPX4, and glutathione, another on ferritin sequestration or turnover, and another on cellular or extracellular iron disposal. The common predicted phenotype is functional survival despite continued environmental iron and hypoxic stress, not uniform expression of a single marker.

Within this framework, loss-of-function alterations in *ARID1A* and loss of ARID1A protein expression retain their established roles as early oncogenic events. We additionally propose that ARID1A deficiency may modify the fitness landscape by changing basal glutathione metabolism or ferroptotic vulnerability. These roles are not mutually exclusive. The specific prediction that ARID1A deficiency intensifies early ferroptotic attrition and increases dependence on compensatory pathways is testable and may be supported or refuted independently of the broader selection model.

## 3 Biological rationale

### 3.1 Origin of the coupled iron–hypoxia environment

Repeated cyclic bleeding into an endometriotic cyst introduces erythrocytes and heme into an enclosed cavity with limited drainage. Progressive hemolysis and retention of blood-derived products can generate high concentrations of free or weakly liganded iron, which catalyze Fenton chemistry and promote lipid peroxidation, protein damage, and oxidative DNA injury (4–7, 19). Unlike an acute hemorrhage that is cleared and repaired, the cystic environment may be renewed over many menstrual cycles, creating a prolonged exposure history.

Oxygen availability may simultaneously be reduced by restricted diffusion, inflammatory and cellular oxygen consumption, and fibrotic remodeling. Takenaka et al. reported a mean cyst-fluid pO_2_ of approximately 22 mmHg in nine ovarian cyst samples together with free iron, iron deposition, and CA9 expression in endometriotic tissue (13). The same study suggested that hypoxia can stabilize iron regulatory protein 2 despite iron excess, illustrating one mechanism through which oxygen and iron regulation may intersect. Because the sample was small and focused largely on stromal cells, these observations require replication and do not establish the oxygen state experienced by epithelial precursor cells over time.

The initiating premise of the model is therefore ecological co-occurrence rather than proven molecular synergy. Iron-related oxidative stress and hypoxia need to be demonstrated in the same lesions and, ideally, in spatial proximity to the epithelial populations being evaluated. Their coexistence is necessary for the proposed coupled selection landscape but is not sufficient to prove that selection has occurred.

### 3.2 A putative ferroptosis-related selection filter

Ferroptosis is mechanistically plausible in this setting because redox-active iron and phospholipid peroxidation are defining requirements for this form of cell death (8–10). Cells with inadequate capacity to detoxify lipid hydroperoxides or control metabolically available iron would be expected to have lower survival under sustained iron-related stress. Conversely, cells that maintain GPX4 activity, glutathione availability, favorable lipid composition, or restricted labile iron may persist more effectively.

Nevertheless, the existence of a ferroptosis-related selection filter in human precursor epithelium has not been shown. Tissue accumulation of 4-hydroxynonenal (4-HNE), expression of SLC7A11 or GPX4, and iron deposition can indicate exposure or response, but none independently demonstrates ferroptotic death or functional tolerance. In viable models, ferroptosis attribution requires concordant evidence of lipid peroxidation, iron dependence, and rescue by ferroptosis-inhibiting interventions supported by orthogonal pharmacological or genetic evidence. In tissue, the strongest achievable evidence will likely be a composite spatial pattern linked to functional findings in matched experimental systems.

Hypoxia may modify this process, but its direction cannot be assumed. In some systems, HIF-1α-mediated signaling supports ferroptosis resistance through metabolic reprogramming and SLC1A1 activation (11). In other settings, hypoxia-related nutrient limitation, mitochondrial dysfunction, or altered lipid metabolism could increase stress or suppress proliferation without conferring a survival advantage. The model therefore predicts context-dependent contributions of iron and oxygen availability to relative fitness through main effects, an interaction, or both, rather than a universal rule that hypoxia protects cells from ferroptosis.

Functional studies in established OCCC models indicate that ferroptotic susceptibility is condition dependent rather than uniform. Cysteine deprivation induced oxidative-stress-dependent necrosis and ferroptosis in glycolytic OCCC models, whereas respiration-dependent models predominantly underwent apoptosis related to impaired iron–sulfur-cluster biogenesis (20). In a separate study, eribulin-induced OCCC cell death was attenuated by ferrostatin-1 and deferoxamine and was accompanied by increases in intracellular iron and lipid peroxidation (21). These studies demonstrate that ferroptosis can contribute to OCCC cell death under defined perturbations, but they neither establish a precursor-stage selection history nor imply uniform sensitivity across OCCC.

### 3.3 Adaptive regulation of the labile iron pool

A central refinement of the model is the distinction between total environmental iron and the intracellular labile iron pool (LIP). The LIP is the metabolically available, redox-active fraction most directly capable of catalyzing lipid peroxidation. An iron-rich extracellular environment does not necessarily produce a persistently high intracellular LIP because cells can regulate iron uptake, sequestration, trafficking, use, recycling, and export (10).

We therefore propose that selected precursor populations may acquire an adaptive iron-handling state that constrains the LIP despite continued exposure to iron-rich cyst contents. Candidate mechanisms include altered transferrin receptor-mediated uptake; sequestration by ferritin heavy and light chains; regulated ferritin turnover, including context-dependent modulation of nuclear receptor coactivator 4 (NCOA4)-mediated ferritinophagy; allocation of iron to mitochondria or other compartments; ferroportin-mediated cellular export; and extracellular-vesicle-mediated disposal of iron-rich ferritin. Enhanced ferritinophagy can release stored iron and promote ferroptosis (22); adaptive regulation therefore concerns its balance with iron sequestration, use, and export, rather than assuming that increased ferritinophagy is protective. Different clones may reach a similar functional state through different combinations of these mechanisms, and a single directional marker pattern should not be expected in every lesion.

Battaglia et al. demonstrated that the ovarian cancer cell lines COV318 and PEO4 limited LIP accumulation after erastin exposure by releasing iron-rich ferritin through CD63-positive extracellular vesicles; inhibition of extracellular-vesicle biogenesis or CD63 depletion restored intracellular iron accumulation, lipid peroxidation, and erastin sensitivity (23). This study provides proof of principle that extracellular ferritin disposal can contribute to ferroptosis resistance in established ovarian cancer cells. It does not demonstrate that the same pathway operates in OCCC, endometriotic epithelial cells, or human precursor lesions. Extracellular-vesicle-mediated ferritin export is therefore incorporated here as a candidate adaptive route requiring direct validation, not as an established component of OCCC carcinogenesis.

### 3.4 Antioxidant and hypoxia-responsive metabolic adaptation

Control of the LIP is likely to cooperate with antioxidant and metabolic programs. In some solid-tumor systems, HIF-1α can promote ferroptosis resistance through a lactate-associated, pH-dependent mechanism and through increased *SLC1A1* transcription with enhanced cystine uptake (11). Oxidative stress can also activate nuclear factor erythroid 2-related factor 2 (NRF2), which transcriptionally regulates *SLC7A11* in several cellular contexts and may enhance cystine uptake for glutathione synthesis (24). These pathways could converge functionally on suppression of lethal lipid peroxidation, but their coordinated operation in endometriotic precursor epithelium has not been demonstrated.

HIF-1α-mediated induction of GLUT1 and glycolytic reprogramming may contribute to glycogen accumulation. In OCCC models, HNF-1β promotes glucose uptake and glycolytic activity (25) and may reinforce the glycogen-rich metabolic phenotype characteristic of OCCC (3, 12, 25). We do not propose a rigid linear pathway in which HIF-1α necessarily induces HNF-1β or in which glycogen accumulation itself causes ferroptosis tolerance. Rather, HIF-1α, NRF2, HNF-1β, and iron-handling pathways are candidate components of a coordinated or convergent survivor state. Their temporal order and causal relationships should be determined experimentally.

This formulation separates two linked but distinct predictions. The first is functional survival under iron–hypoxia stress. The second is acquisition of the glycogen-rich, clear-cell metabolic phenotype. These outcomes may co-emerge because they share upstream metabolic pressures, but either could occur without the other. Failure to demonstrate a direct link between glycogen accumulation and ferroptosis tolerance would narrow, rather than necessarily invalidate, the core selection hypothesis.

### 3.5 ARID1A as an oncogenic driver and putative modifier of selection

Loss-of-function mutations in *ARID1A* and loss of ARID1A protein expression are recurrent early events in endometriosis-associated OCCC and contribute to carcinogenesis through chromatin-regulatory and transcriptional mechanisms (3, 26). These readouts should not be treated as biologically interchangeable: *ARID1A* loss-of-function mutations can be detected in ovarian endometriosis despite retained ARID1A protein expression, consistent with a context-dependent two-hit process (27). The present model does not replace the established driver interpretation. Instead, it proposes an additional, context-dependent role for ARID1A deficiency in shaping vulnerability to environmental stress.

Ogiwara et al. showed that ARID1A-deficient cancer cells can have reduced *SLC7A11* expression and basal glutathione levels and can be selectively vulnerable to inhibition of glutathione metabolism (28). Extrapolating this finding to endometriotic precursor epithelium, we hypothesize that ARID1A deficiency may increase early susceptibility to lipid-peroxidative stress and create greater dependence on compensatory antioxidant or iron-handling pathways. Under repeated stress, this could increase attrition among ARID1A-deficient cells while enriching those that acquire adequate compensation.

This selection-modifying role remains inferential. ARID1A deficiency could increase cell loss, alter which clones survive, increase dependence on a particular compensatory pathway, or have no measurable effect in the endometriotic context. Failure to observe increased acute ferroptotic susceptibility or greater survivor dependence would challenge the corresponding directional prediction. A reproducible opposite effect would refute that prediction while remaining compatible with a different selection-modifying role; neither outcome would by itself invalidate the broader environmental-selection model.

### 3.6 Potential contribution to histotype divergence

Endometriosis is associated with both OCCC and endometrioid carcinoma, yet the two histotypes differ in morphology and molecular profile (29). The shared cyst microenvironment is therefore unlikely to specify histotype on its own. As a secondary extension of the model, environmental selection may interact with pre-existing or emerging lineage-defining genetic and epigenetic states to bias divergent phenotypes. Comparative spatial studies of precursors associated with the two histotypes could test whether their metabolic and iron-handling states differ, without presuming a single lineage pathway.

This proposal is more speculative than the core iron–hypoxia selection hypothesis and is not required for the core model to be valid. Genetic background, epigenetic state, immune and stromal interactions, and anatomical context may all influence lineage outcome. Detailed testing of histotype divergence should therefore follow, rather than precede, validation of the environmental and ferroptosis-related selection premises.

## 4 Evaluation of the proposed model

### 4.1 Evidence-supported foundations

Several foundations of the model are supported by published evidence. Endometriotic cyst fluid is iron rich and capable of producing oxidative stress and DNA damage (4–7, 19). Low oxygen tension, CA9 expression, and abnormal iron regulation have been reported in ovarian endometriotic cysts (13). HIF-1α and GLUT1 expression increase during progression toward OCCC (12). Core mechanisms of ferroptosis, including dependence on redox-active iron, phospholipid peroxidation, glutathione, and GPX4, are well established (8–10). HIF-1α/SLC1A1- and NRF2/SLC7A11-associated protective pathways have been demonstrated in non-precursor experimental systems (11, 24), ARID1A-deficient cancer cells can exhibit glutathione vulnerability (28), defined OCCC models can undergo ferroptosis under selected perturbations (20, 21), and established ovarian cancer cell lines can use CD63-positive extracellular vesicles to dispose of iron-rich ferritin (23).

These observations support biological plausibility but arise from different tissues, disease stages, and experimental contexts. They do not collectively prove the proposed sequence in human precursor epithelium.

### 4.2 Undemonstrated mechanistic links

The following links remain unverified: direct ferroptotic death of susceptible epithelial cells in endometriotic precursor lesions; a reproducible contribution of both iron and oxygen context to precursor-cell fitness; repeated exposure followed by clonal or cellular-population enrichment attributable to differential survival or expansion; coordinated precursor-stage regulation of the LIP; extracellular-vesicle-mediated ferritin disposal by endometriotic or atypical epithelial cells; and modification of this process by *ARID1A* mutation or ARID1A protein-loss status. Durable phenotypic adaptation after washout is a related but distinct outcome and would not alone establish selection. The proposed connection between the survivor state and glycogen-rich clear-cell morphology is also inferential.

Importantly, coexistence of iron and hypoxia is not equivalent to mechanistic coupling, and expression of stress-response proteins is not equivalent to selection. Cross-sectional tissue studies can identify spatial and stage-associated patterns, but repeated-exposure functional models are required to demonstrate differential survival, persistence, and causality.

### 4.3 Falsifiability and model boundaries

The core model would gain support if four conditions were met: iron-related oxidative stress and hypoxia coexist in the relevant precursor niche; a prespecified 2 × 2 factorial analysis shows that both iron and oxygen context contribute to relative fitness through main effects, an interaction, or both; repeated exposure produces reproducible enrichment or depletion attributable to differential survival or expansion rather than state switching alone; and ferroptosis-inhibiting or ferroptosis-directed perturbations, interpreted with orthogonal evidence, modify at least part of the selective cell loss and associated enrichment or depletion. A supra-additive interaction is not required. Evidence for environmental co-occurrence alone would establish exposure but not selection.

The model would be weakened if iron and hypoxia do not coexist reproducibly; if factorial analyses show no reproducible contribution from one stress axis and no iron-by-oxygen interaction across biologically relevant exposure ranges; if repeated exposure produces no population enrichment or depletion attributable to differential survival or expansion; or if cell loss is not modifiable by ferroptosis-inhibiting interventions under conditions that otherwise satisfy orthogonal criteria for ferroptosis. Absence of supra-additivity alone would not refute the model. Durable changes that occur broadly without differential enrichment would instead support persistent adaptation, whereas findings dominated by other death pathways would narrow the proposed mechanism. The model may also be narrowed rather than rejected; for example, selection could be supported while the directional ARID1A-vulnerability or glycogen-link subhypotheses are refuted.

### 4.4 Specific conceptual contribution

The novelty of this framework does not lie in asserting previously unrecognized interactions among iron metabolism, hypoxia, oxidative stress, and ferroptosis. It lies in applying these interconnected processes to a precursor-stage evolutionary question: whether persistent iron-rich and hypoxic conditions change the relative fitness of epithelial populations across the endometriosis–atypical endometriosis–OCCC continuum. **Figure 2** contrasts this precursor-stage selection question with a simplified overview of the interconnected biology commonly studied in other experimental contexts.

**Figure 2:**
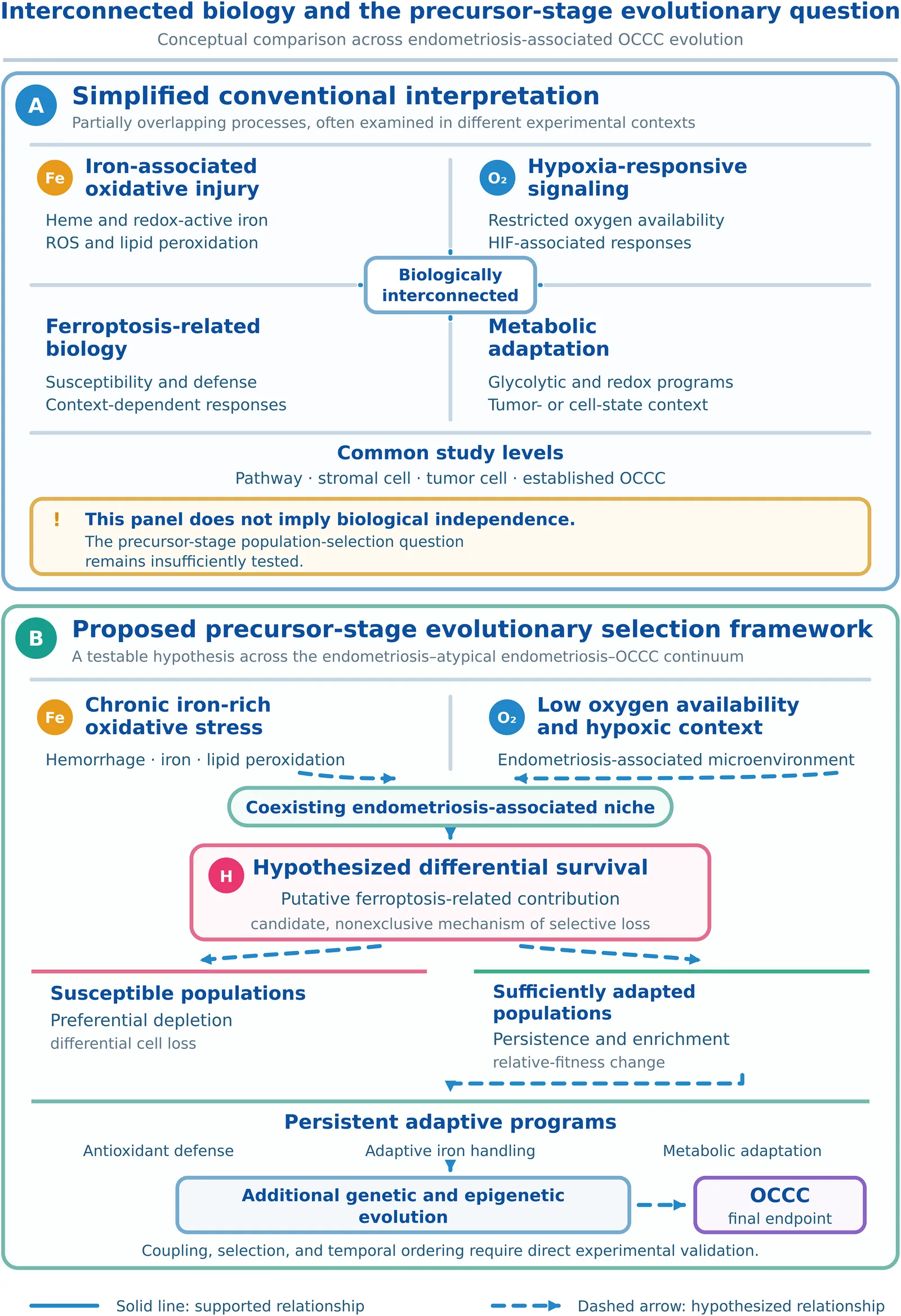
Simplified conventional interpretation and the proposed precursor-stage evolutionary selection model. Panel **(A)** presents a simplified conventional interpretation in which iron-associated oxidative injury, hypoxia-responsive signaling, and ferroptosis-related biology are considered as partially overlapping but separately investigated features of endometriosis or established ovarian clear cell carcinoma (OCCC). This panel is not intended to represent a universally accepted model or to imply that interactions among these processes have not previously been recognized. Panel **(B)** presents the hypothesis proposed in this article. Chronic iron-rich oxidative stress and a hypoxic microenvironment are placed upstream of overt malignant transformation and are proposed to alter the relative fitness of epithelial precursor populations across the endometriosis–atypical endometriosis–OCCC continuum. A putative ferroptosis-related selection filter may contribute to differential cell loss and survivor enrichment, followed by persistent antioxidant, iron-handling, and metabolic adaptation. Dashed arrows identify hypothesized causal or evolutionary relationships. The proposed coupling, selection process, and temporal ordering remain to be validated experimentally.

## 5 Experimental predictions

**Table 1** summarizes the rationale, experimental approach, expected finding, and alternative interpretation for the four experimental predictions.

**Table 1:** Summary of the four experimental predictions in the revised iron–hypoxia selection model.

Prediction	Rationale	Experimental approach	Expected finding	Alternative interpretation/falsification
Prediction 1: Coexisting exposures (§5.1)	Joint selection requires iron-related oxidative stress and hypoxia to coexist in the same precursor environment.	Measure oxygen promptly in fresh cyst fluid, alongside iron and lipid-peroxidation indices; pair fluid measurements with spatially localized epithelial findings and comparator lesions.	Low oxygen coexists with iron-related oxidative stress. Current cyst-fluid iron need not increase monotonically across disease stages.	Failure to detect co-occurrence weakens the exposure premise. Co-occurrence alone cannot demonstrate ferroptosis or selection.
Prediction 2: Preinvasive metabolic programs (§5.2)	Adaptation contributing to precursor evolution should emerge before invasive carcinoma.	Compare typical and atypical endometriosis and OCCC using spatial HIF-1α, CA9, GLUT1, HNF1β, and glycogen staining with diastase controls; relate epithelial findings to local iron and oxidative injury.	Hypoxia-responsive or glycogen-associated programs are spatially enriched or progressively activated in precursor epithelium.	Changes confined to invasive cancer favor a tumor-stage explanation. Static glycogen staining does not establish metabolic flux or causality.
Prediction 3: Composite iron-handling states (§5.3)	Single antioxidant or ferritin markers cannot establish functional ferroptosis tolerance.	Combine iron uptake, storage, turnover, export, antioxidant, and lipid-peroxidation measures with epithelial localization and functional labile-iron assessment. Test vesicle-mediated ferritin disposal with validated vesicle assays.	Precursor populations show coordinated survival-associated states with a constrained labile iron pool despite iron exposure; adaptive routes may vary.	Cancer-only changes or lack of functional concordance weaken the premise. Similar profiles in nonatypical lesions limit specificity; expression patterns alone do not establish selection.
Prediction 4: Persistent selection and ARID1A modulation (§5.4)	Acute susceptibility does not establish selection; ARID1A deficiency may alter vulnerability and survivor enrichment.	Use isogenic models, factorial iron/hypoxia exposure, repeated sublethal cycles, lineage tracking, washout, rechallenge, and orthogonal ferroptosis assays. Prespecify outcomes, replication, and statistical analysis.	Both exposure axes contribute through main effects and/or interaction, with persistent differential clonal enrichment. The directional ARID1A hypothesis predicts greater initial vulnerability with compensatory survivor enrichment.	State switching alone does not establish selection. A missing contribution from one axis, without interaction, weakens the coupled model; lack of supra-additivity alone does not. Opposite ARID1A effects challenge the directional subhypothesis.

These are prospective tests, not reported experimental results. For detailed controls and evidentiary limits, see Sections 5.1–5.4. OCCC, ovarian clear cell carcinoma; HIF-1α, hypoxia-inducible factor 1 alpha; CA9, carbonic anhydrase 9; GLUT1, glucose transporter 1; HNF1β, hepatocyte nuclear factor 1 beta; ARID1A, AT-rich interaction domain 1A.

### 5.1 Prediction 1: iron-related oxidative stress and hypoxia coexist within the same cyst microenvironment

#### 5.1.1 Rationale

The proposed selection landscape requires simultaneous or recurrent exposure to both stress axes.

#### 5.1.2 Experimental approach

Fresh cyst fluid should be collected using a standardized protocol that minimizes air exposure and processing delay. Measurements should include oxygen tension, free or labile iron, total iron and ferritin, and lipid-peroxidation products such as 4-HNE or complementary oxidative-lipid markers. Appropriate comparators should include non-endometriotic benign cyst fluids and, where available, endometriosis-associated carcinoma fluids. Paired tissue should be evaluated for iron deposition and hypoxia-related markers, allowing cyst-fluid findings to be linked spatially to the epithelial compartment.

#### 5.1.3 Expected finding

Endometriotic cysts should reproducibly demonstrate coexisting low oxygen tension, elevated iron burden, and lipid-peroxidative stress linked to the epithelial compartment. Differences across typical, atypical, and carcinoma-associated lesions should be tested without assuming a monotonic increase. Lower total iron concentrations have been reported in carcinoma-associated cyst fluid than in benign endometriotic cyst fluid (6); measurements at sampling therefore should not be equated with cumulative precursor exposure.

#### 5.1.4 Alternative interpretation

Failure to reproduce coexisting iron and hypoxia would weaken the initiating premise. Demonstration of co-occurrence without epithelial adaptation would support environmental exposure but not the proposed selection process.

### 5.2 Prediction 2: hypoxia-responsive and glycogen-associated programs emerge before invasive carcinoma

#### 5.2.1 Rationale

If metabolic adaptation contributes to precursor survival, relevant changes should be detectable in atypical endometriosis rather than appearing only after invasion.

#### 5.2.2 Experimental approach

Spatial analysis should compare typical endometriosis, atypical endometriosis adjacent to OCCC, noncontiguous endometriosis without histological atypia from the same ovary when available, and established OCCC. HIF-1α, CA9, GLUT1, HNF-1β, and periodic acid-Schiff (PAS) staining with matched diastase digestion controls should be evaluated in the epithelial compartment and interpreted in relation to local iron deposition and oxidative injury.

#### 5.2.3 Expected finding

OCCC-associated precursor lesions should show spatial enrichment or stepwise activation of hypoxia-responsive and glycogen-associated features before invasive carcinoma.

#### 5.2.4 Alternative interpretation

If these changes are confined to established OCCC, they are more likely to represent tumor-stage metabolic adaptation than precursor selection. Even diastase-sensitive PAS staining is a static measure and cannot establish metabolic flux or a causal relationship with ferroptosis tolerance.

### 5.3 Prediction 3: precursor lesions exhibit composite iron-handling and lipid-peroxidation states rather than a uniform single-marker signature

#### 5.3.1 Rationale

Increased SLC7A11, GPX4, or ferritin heavy chain 1 (FTH1) expression alone may reflect general oxidative stress and cannot establish functional ferroptosis tolerance. The model predicts coordinated regulation of antioxidant defense, intracellular iron availability, and lipid peroxidation.

#### 5.3.2 Experimental approach

A composite tissue panel should include SLC7A11 and GPX4; transferrin receptor/CD71; ferritin heavy and light chains; NCOA4; ferroportin/SLC40A1; acyl-CoA synthetase long-chain family member 4 (ACSL4); and 4-HNE. These markers should be evaluated with HIF-1α, ARID1A protein expression, HNF-1β, PAS staining with matched diastase digestion controls, and local iron deposition. *ARID1A* mutation status and ARID1A protein loss should be recorded separately because they are not biologically equivalent. Ferritin localization and heterogeneity may be more informative than expression intensity alone. Direct measurements of LIP and lipid peroxidation should be performed in fresh epithelial cells, organoids, or other viable models because fixed tissue cannot provide a dynamic LIP measurement.

Extracellular-vesicle-associated ferritin should be assessed in cyst fluid or conditioned medium using validated vesicle isolation and characterization, particle quantification, vesicle markers, and ferritin cargo analysis. Tissue CD63 expression alone should not be interpreted as evidence of extracellular ferritin export.

#### 5.3.3 Expected finding

OCCC-associated precursor populations should display a coordinated state compatible with survival under iron and lipid-peroxidative stress. Different lesions may use different routes, but functional models should demonstrate that survivor populations maintain a lower or more tightly controlled LIP than susceptible populations exposed to the same extracellular iron burden.

#### 5.3.4 Alternative interpretation

Restriction of candidate patterns to invasive carcinoma, or lack of corresponding functional differences in LIP, lipid peroxidation, or ferroptosis susceptibility, would weaken the proposed precursor iron-handling phenotype. Similar patterns in noncontiguous, non-atypical endometriosis would weaken their specificity for OCCC-associated precursors, but would not alone exclude early adaptation or selection. Marker distributions cannot establish selection without functional evidence of differential survival or expansion.

### 5.4 Prediction 4: repeated sublethal iron–hypoxia exposure produces persistent selection, and ARID1A deficiency modifies the outcome

#### 5.4.1 Rationale

Acute exposure tests susceptibility, whereas the central hypothesis concerns evolutionary selection over repeated stress cycles.

#### 5.4.2 Experimental approach

Ideally, isogenic ARID1A-intact and ARID1A-deficient endometriosis-derived epithelial cells or organoids should be used; established OCCC models may provide complementary but later-stage evidence. Experiments should use a 2 × 2 factorial design comprising untreated, iron-only, hypoxia-only, and combined iron–hypoxia conditions. The analysis should prespecify iron and oxygen main effects and their interaction; absence of supra-additivity should not be treated as experimental failure. Stress intensity should be pre-titrated to produce measurable attrition while preserving sufficient survivors for repeated cycles. Future validation studies should prespecify primary outcomes, biological replication, sample-size justification, and statistical analyses appropriate to the factorial and repeated-measures design, with statistical input during study planning.

An initial acute module should quantify differential susceptibility using cell death, lipid peroxidation, LIP, clonogenic survival, and rescue by ferroptosis-inhibiting interventions or iron chelation, supported by orthogonal pharmacological or genetic evidence. This module can test whether ARID1A deficiency increases acute ferroptotic vulnerability but cannot demonstrate selection.

The principal selection module should expose populations to repeated sublethal cycles followed by recovery and washout. Lineage barcoding or an equivalent tracking strategy should link baseline clones or states to longitudinal survival, division, and population frequencies. Replicate cultures and untreated controls undergoing the same passaging schedule are needed to distinguish differential fitness from state switching, drift, or passage-related bottlenecks. After washout, survivor populations should be rechallenged with combined stress and defined ferroptosis inducers. Outcomes should include clonal composition, persistence of altered susceptibility, LIP, lipid peroxidation, mitochondrial redox state, SLC7A11/GPX4 and NRF2 activity, ferritin synthesis and turnover, NCOA4, ferroportin, iron compartmentalization, and extracellular-vesicle number and ferritin cargo.

#### 5.4.3 Expected finding

Across the factorial conditions, iron and oxygen context should each make a reproducible contribution to relative fitness through main effects, an interaction, or both; the combined condition need not be the most cytotoxic. Under repeated exposure, identifiable clones or cellular populations should be reproducibly enriched or depleted through differential survival or expansion. State-frequency changes explained by switching alone, including durable remodeling after washout, would support adaptation rather than demonstrate selection. The prespecified ARID1A subhypothesis predicts greater initial attrition in ARID1A-deficient populations and stronger dependence of their survivors on compensatory antioxidant or iron-handling pathways.

#### 5.4.4 Alternative interpretation and falsification

Transient transcriptional responses that disappear after washout without reproducible population enrichment or depletion would indicate reversible adaptation rather than selection. If either iron or oxygen context makes no reproducible contribution to relative fitness and no interaction is observed across biologically relevant exposure ranges, the coupled-selection model would be weakened; absence of supra-additivity alone would not refute it. Failure of ferroptosis-inhibiting interventions to modify cell loss, together with failure to meet orthogonal criteria for ferroptosis, would favor another death mechanism. No reproducible effect of ARID1A functional status would weaken its proposed modifying role. An effect opposite to the prespecified increase in vulnerability or survivor dependence would refute that directional prediction, while leaving other modifying effects and the broader selection model open to evaluation.

## 6 Discussion

### 6.1 Conceptual implications

The proposed framework shifts attention from how established OCCC withstands oxidative stress to how epithelial precursor populations persist long enough to contribute to carcinoma. It complements rather than replaces mutation-centered models. Genetic and epigenetic alterations create diversity, while a persistent microenvironment can change the relative fitness of those variants. In this view, malignant evolution reflects both the generation of cellular variation and the ecological filtering of that variation.

The model also reframes iron exposure. Iron is not only a mutagenic or cytotoxic agent but a potential determinant of which cellular states remain viable. The relevant variable is not total extracellular iron alone, but the relationship between environmental iron burden and the intracellular LIP. This distinction accommodates apparently paradoxical observations in which ovarian cancer cells remain iron dependent yet avoid ferroptosis by altering ferritin storage, turnover, trafficking, or extracellular disposal.

Ferroptosis is deliberately positioned as a candidate mechanism rather than a foregone conclusion. The iron-rich, lipid-peroxidative setting makes it plausible, but human precursor tissue cannot currently establish ferroptotic death by marker expression alone. The model therefore stands or falls on functional evidence from repeated-exposure systems linked back to spatial patterns in human lesions. This evidentiary architecture is important because it prevents a collection of stress markers from being mistaken for proof of evolutionary selection.

### 6.2 Evolutionary trade-offs and therapeutic hypotheses

Repeated survival under iron-related and hypoxic stress may produce an evolutionary trade-off. The adaptations that permit precursor cells to survive could later become dependencies that restrict the viable metabolic states of established OCCC. This does not imply that all OCCC tumors are uniformly ferroptosis resistant or uniformly ferroptosis sensitive. Indeed, functional OCCC studies show different death responses according to metabolic state and perturbation (20, 21). Different tumors may depend on different protective routes, including the SLC7A11-GPX4-glutathione axis, NRF2-associated antioxidant signaling, ferritin synthesis or turnover, maintenance of a restricted LIP, ferroportin-mediated export, or extracellular-vesicle-mediated ferritin disposal.

This heterogeneity creates testable therapeutic hypotheses. In the Battaglia et al. ovarian cancer models, blocking extracellular-vesicle biogenesis or CD63 restored LIP accumulation and ferroptosis sensitivity (23). Eribulin-induced OCCC cell death was attenuated by ferroptosis-inhibiting interventions in preclinical models (21), and Class IV ferroptosis inducers have been proposed as a means of disrupting iron homeostasis and increasing the LIP in epithelial ovarian cancer models (30). These findings identify experimental strategies for probing pathway dependence, but they do not establish that the dependencies originated during precursor selection or justify clinical treatment.

Therapeutic studies should therefore be biomarker stratified and mechanistically matched. A tumor dependent on extracellular ferritin disposal may respond differently from one dependent on GPX4 activity or reduced iron uptake. Normal endometriotic epithelium and other tissues may share some of these protective pathways, creating potential toxicity. Bioavailability, delivery specificity, and systemic iron effects are additional barriers to translation (30). At present, the therapeutic value of the model lies in generating dependency-based experiments rather than recommending ferroptosis-modulating therapy.

### 6.3 Limitations and research priorities

This article presents a conceptual model and contains no new experimental data. Most supporting observations derive from cross-sectional human tissue, stromal-cell studies, established cancer cell lines, or non-OCCC tumor systems. Extrapolation to epithelial precursor evolution is therefore substantial. The reported cyst-fluid oxygen measurements were obtained from a small sample, and a single intraoperative measurement cannot reconstruct years of fluctuating exposure. Atypical endometriosis is uncommon, heterogeneous, and frequently identified adjacent to carcinoma, where reverse influence from an established tumor and field effects may confound temporal inference.

Marker-based tissue studies have additional limitations. SLC7A11, GPX4, FTH1, ACSL4, 4-HNE, CD71, NCOA4, ferroportin, and CD63 are not individually specific for ferroptosis tolerance or selection. Cross-sectional gradients may reflect tumor proximity, generalized oxidative stress, or altered differentiation. Direct LIP, lipid-peroxidation dynamics, ferroptosis rescue, and persistent survivor behavior must therefore be assessed in viable models. Likewise, lineage barcoding can demonstrate clonal enrichment *in vitro* but cannot fully reproduce the immune, stromal, endocrine, and mechanical context of an endometriotic cyst.

The most efficient testing sequence is hierarchical. First, paired cyst-fluid and tissue studies should establish whether iron-related oxidative stress and hypoxia coexist reproducibly in the epithelial niche. Second, spatial profiling should determine whether composite iron-handling and metabolic states appear in atypical endometriosis before invasion. Third, repeated-exposure organoid or epithelial models should test persistence, clonal enrichment, ferroptosis dependence, and modification by ARID1A functional status. Only after these steps should therapeutic dependencies be examined systematically in molecularly stratified OCCC models.

This hierarchy allows partial revision of the theory. The environmental premise, ferroptosis mechanism, ARID1A modifier, metabolic-morphology link, and the more speculative histotype-divergence extension can each be evaluated separately. The model is therefore not a single all-or-none narrative but a set of linked propositions whose supported components can be retained while unsupported components are discarded.

## 7 Conclusions

We propose that chronic iron-rich oxidative stress and hypoxia may alter the relative fitness of epithelial populations during the transition from endometriosis to OCCC. Ferroptosis is a candidate, but not necessarily exclusive, mechanism of selective cell loss. Populations capable of controlling the LIP, suppressing lethal lipid peroxidation, or activating compensatory antioxidant and metabolic programs may preferentially persist under repeated stress.

The distinctive contribution of this hypothesis is not the identification of a previously unknown molecular interaction. It is the application of interconnected iron, hypoxia, redox, and ferroptosis biology to an evolutionary selection model across the endometriosis–atypical endometriosis–OCCC continuum. *ARID1A* alteration remains an oncogenic driver event, and ARID1A deficiency is additionally proposed as a context-dependent modifier of stress vulnerability, not as an alternative to its established driver role.

The framework generates separable and falsifiable predictions. Cyst-fluid and spatial studies can establish the environmental and precursor-stage context, while repeated sublethal exposure, washout, rechallenge, and lineage tracking can distinguish acute susceptibility, differential enrichment, and durable adaptation. The model would be weakened if coupled exposure is not reproducible, if one stress axis makes no reproducible contribution to relative fitness and no iron-by-oxygen interaction is observed across biologically relevant conditions, if repeated exposure produces no enrichment or depletion attributable to differential survival or expansion, or if the selective cell loss is not modifiable through ferroptosis-related mechanisms supported by orthogonal evidence. Lack of supra-additivity alone would not refute the model. It should therefore be regarded as a generative framework that can be supported, narrowed, reformulated, or rejected through experimental investigation.

## Data Availability

The original contributions presented in the study are included in the article/supplementary material. Further inquiries can be directed to the corresponding author.
